# Inflammation of the Neck of the Bladder

**Published:** 1888-05

**Authors:** Jno. Thad. Johnson

**Affiliations:** Atlanta


### INFLAMMATION OF THE NECK OF THE BLADDER.

BY JNO. THAD. JOHNSON, M. D.

There is a certain stage of the disease in which our old-time friends, copaiba, cubebs, oil of sandal wood, oil of turpentine, and possibly some others of the class, do undoubtedly exert a beneficial effect. In the subacute stage, and sometimes when it has become chronic, the distressing symptoms are modified by these agents singly or in combination. Indeed it may be possible to effect a cure with them, if aided by rest and hygiene at the proper time.

Alkalies, in various forms and combinations are generally prescribed. They often afford relief—more in this condition than in general chronic vesical catarrh, when the urine is probably of itself already too alkaline. For the distinctly alkali effect, the liquor potassæ answers well.

The salts of potassa, lithia, ammonia, and other similar agents, are generally prescribed, and often with seeming good effect. The benzoate of ammonia is a favorite with me ; this, dissolved in camphor water, and a minute portion of morphia added to it, has seemed to me to afford relief, when distress is marked, more promptly and certainly than others. Tincture of chloride of iron undoubtedly is sometimes useful. Tincture of cantharides has long been employed in such affections, often combined with the chloride of iron tincture. I am not satisfied that it possesses any reliable virtue. It is not beyond its power to render the condition worse rather than better. The iron does not do this ; for this reason, as well as for its positive virtue, I think it preferable. This is better prescribed when the disease has passed beyond its subacute stage.

There is yet another class of remedies requiring notice, consisting of buchu, uva ursi, pareira brava, triticum repens, hydrangea, etc. These are looked upon as having a direct effect, mostly an astringent one, upon the mucous membrane or the urinary tract. Like most of such remedies, their actions cannot be deemed reliable ; yet as they do sometimes appear to have the desired control over function and secretion, and as they seem but little capable of doing an injury, we are justified in testing their efficacy in any given case. They may be administered in fluid extract or infusion. I generally have an infusion made of several of these agents, together with hops, and then adding bicarbonate of soda. It is a kind of a shot-gun medication from which I have but little to fear, even if I cannot allow myself much latitude of hope in its therapeutical efficacy.

Lastly, let us consider local measures. It is from this method of treatment that we may look for the best results. But to reach a successful result, we must be more careful of our remedies, and more exact in applying them, than has been the general custom.

In making these applications, I hold that it is best to apply them directly to the surface diseased, and to no other portion, so far as may be possible.

Already I have shown that very often the patient is directed to continue his injections, and with the same instrument that served for medicating that part of the canal which lies in front of the cut-off muscle. Its range is too short for the work now in hand; it cannot be depended upon for forcing the fluid beyond the compressor muscle. This difficulty is often met by passing an instrument into the bladder, and then filling the whole bladder with the solution selected. I wish here to enter my objection against the adoption of this as a standard treatment. I object to it because our fluid comes in contact with an extent of healthy membrane much larger than that which is diseased; and of all tissues, this is among the most intolerant of meddlesome medication. Certainly a diseased bladder must sometimes be treated by direct application. This cannot be omitted in chronic catarrh; but in acute cystitis, general cystitis, to say the best of it, local medication must be done only with great caution. And when the membrane is not inflamed, certainly it is far worse than useless to medicate it. Such a step may serve to light up a disease where none had existed.

The deduction I would make from the principles and argument just laid down, is the necessity of confining our topical medication to the part diseased, as accurately as may be. As it is necessary to do more than treat the anterior urethra by aid of the ordinary syringe, so, on the other hand, it is unnecessary to go to the other extreme, and fill an unoffending bladder with our remedy merely because the surface just posterior to the compressor urethræ requires treatment. I have known a number of patients whose condition had been made worse rather than bettered by a non-appreciation of these truths. I thus emphasize it, because, as a rule, neither book writers nor the profession generally, lay stress upon what I deem the necessities of the condition described.

To place our remedies accurately upon the diseased region, some instrument is required that will enable us to know just when its opening has reached the bladder neck. The tube, or catheter, may be of gum, mettal or hard rubber. Several forms of instruments have been specially devised for this medication. Some of those originally intended for spermatorrhœa treatment will serve the present purpose. So that the indications just laid down are met, the form of instrument need not be considered. It is important, however, in the event of our applications being frequently repeated, that the tube be pliable and small, so as not to create irritation by its passage. In this case, the soft rubber catheter with the opening directly in the rounded extremity, is preferable to the ordinary form with the eye on the side. This rare form may be obtained of the instrument makers, and generally enables us to make the application with the utmost ease.

The hard rubber tube, made as a part of the syringe, answers a better purpose for the infrequent and severer applications; and I may add, serves well for the more frequent ones, except for the irritation of passing it. The double current catheter, with large opening, may be used for the topical medication—more for this opening than for its double tube.

To the soft catheter, any syringe that has a capacity of several drams, and that works easily, may be attached. The fountain syringe, or the rubber bag, are not so well adapted to the medication of this special lesion. Whatever tube be used for the urethra, a little care will enable us to discharge the fluid just where needed. While we may not confine it precisely within limits, yet there will not be the harm done of filing an innocent bladder, as is sometimes practiced.

The same remedies are to be used, in the main, as serve to cure an inflammation of the anterior portion of the canal, I have always taught that the weakest solution that will properly accomplish the cure, is the one to be selected. A very good measure to guide us as to the strength, is the very mild degree of smarting that the proper per cent. solution produces. If the pain is violent or long continued, injury instead of benefit will result. This does not apply however, to that stronger, or semi-caustic, application that is occasionally required for the cure of a chronic lesion.

Nitrate of silver, in solution, may be said to serve us in two characters: a very weak one, for regular application, and one much stronger for occasional use—the latter semi-caustic, or highly stimulating in its local action.

In a paper published some years ago, in urging the need for knowing the exact dosage required by the urethra, I made the assertion that the most dilute solutions of this remedy, nitrate of silver, as laid down in the books, are yet stronger than necessary for frequent application to the urethra. The strength proposed, one-eighth to one-sixteenth to the ounce of water, was looked upon by some as a mere trifling. I might adduce, as a further support to my observations, the more recent views carried out under the banner of the germ theory. Even a greater dilution, frequently used, sometimes produces the burning and bloody discharge characteristic of its caustic use.

For most patients, nothing has answered me so well in the treatment of ordinary urethritis. Occasionally, however, a patient is met with in whom it does not prove curative. I have been in the habit of treating one individual in this city for his many gonorrhœas, in whom a one thirty-second grain solution has always caused such burning, bloody discharge, etc, and attended by no benefit, as to demand a change.

I may add, parenthetically, that in several cases of purulent ophthalmia, these dilute solutions, with cleanliness, have made an easy cure after failure of the ordinary procedure. I have not practiced this often enough to offer it to others as a standard treatment; but I may say that I have of late noticed in the journals several statements looking in the same direction.

In the treatment of these urethral affections, the sulphate of copper acts very like the silver nitrate, and in about the same doses. The acetate of lead, one to three grains, is a good remedy. The zinc salts are even better; the acetate, one-half to two grains to the ounce of water; the sulphate, twice as strong; the chloride, half the strength of the acetate. With all of this class, the cases of long standing generally require the stronger solutions. Many other remedies are used, but I know none superior to these.

These remedies are placed in the vesical neck as already indicated, from one to three times daily, provided they can be used so often. As a rule, the surgeon himself can apply it once daily so well as to require nothing further until the next day. The principle to guide us is the same as for the anterior portion of the urethra; only the method of applying the remedies is different. We need not only to be careful to medicate the diseased parts efficiently, but to as carefully avoid that sensitive and extensive surface beyond which would probably be injured by such interference.

While I strenuously advocate the general use of such applications as are generally regarded as very weak, I well know that there is occasional need for a stronger solution of nitrate of silver, of five to twenty grains to the ounce. When the parts have reached that indolent condition in which no improvement is obtained by the ordinary injection, a thorough application of solution just mentioned is indicated. This is better preceded, a few minutes before, by a cocaine solution. A day or two later the milder remedies may be resumed.

Another excellent method of applying the remedies accurately, is by means of a brush and tube. Having saturated the brush with the desired solution (or powder), it is withdrawn into the tube, the apparatus carried to the spot desired, the tube withdrawn sufficiently to uncover the brush, and this thereby brought in contact with the membrane diseased.

I have also used the short urethral suppository for the treatment of this disease. These must be carried down by a proper tube. I have had some good results from them, yet by no means so great as would at first sight appear reasonable to expect.

The majority of these patients who present themselves to me have been already treated by the introduction of sounds. The patient himself, by much practice, has become an expert in the art. I recognize the value of this treatment for the anterior urethra. I well know that it is an excellent method of overcoming an irritability, or super-sensitiveness of the vesical neck. But a catarrh of the neck is another thing. I have more often found injury than benefit to follow the use of the sound. There is not here a stricture to be dilated, as often is the case in the chronic discharge from the anterior portion. There are anatomical elements that serve to render the conditions altogether different. It has sometimes seemed to me astounding how persistently this measure has been resorted to. This generally proves to be true of the cases alluded to in the outset, wherein the real nature, or seat, of the disease has been overlooked.

I may remark here that I believe the introduction of instruments into the neck, and on into the bladder, is too readily undertaken. Its possibilities of evil are not sufficiently considered. When the anterior part of the canal alone requires the passage of an instrument, as in disease, or after operations, we are not justified in passing entirely through it, and into the bladder beyond.

It may seem that I have devoted undue space to the consideration of this disease. But to those who have had opportunity of observing the number of males so affected, the discomfort and ill-health it gives rise to, its long-standing, and the often inadequate treatment relied upon, will appreciate any effort in the direction of its better understanding.

ATLANTA, March, 1888.

